# The epidemiology of HIV and other sexually transmitted infections in African, Caribbean and Black men in Toronto, Canada

**DOI:** 10.1186/s12879-019-3925-3

**Published:** 2019-03-29

**Authors:** LaRon E. Nelson, Wangari Tharao, Winston Husbands, Ting Sa, Nanhua Zhang, Sameer Kushwaha, David Absalom, Rupert Kaul

**Affiliations:** 1 School of Nursing, Yale University, 400 West Campus Drive, New Haven, CT 06477 USA; 2 Centre for Urban Health Solutions, St. Michael’s Hospital, 209 Victoria Street, Toronto, ON M5B 1T8 Canada; 3 Women’s Health in Women’s Hands Community Health Centre, 2 Carlton Street, Suite 500, Toronto, ON M5B 1J3 Canada; 4 Ontario HIV Treatment Network, 1300 Yonge Street, Suite 600, Toronto, ON M4T 1X3 Canada; 5 Division of Biostatistics and Epidemiology, Cincinnati Children’s Hospital Medical Center, 3333 Burnet Ave, Cincinnati, OH 45229 USA; 6 Faculty of Medicine, University of Toronto, 1 Kings College Circle, Toronto, ON M5S 1A8 Canada

**Keywords:** HIV, Sexually transmitted infections, Epidemiology, Black, Health disparities, HIV/STI co-infection, Syphilis, Black Canadian men, Black MSM, Toronto

## Abstract

**Background:**

African, Caribbean, and Black (Black) men account for 16.5% of new HIV diagnoses among men in Ontario. There is substantial evidence that sexually transmitted infections (STIs) are associated with increased likelihood of HIV infection; however, little is known regarding the prevalence of HIV/STI co-infections among Black men in Toronto. Progress has been made in understanding factors contributing to racial/ethnic disparities in HIV between among men who have sex with men (MSM). In this study, we investigate within-racial group patterns of HIV/STI infection between Black MSM and Black men who only have sex with women (MSW).

**Methods:**

A cross-sectional descriptive epidemiological study was conducted with a non-probability sample of Black men recruited from Toronto, Ontario. Audio Computer Assisted Self-Interviews (ACASI) surveys were used to collect demographic and behavioral data. Biological specimens were collected to screen for HIV and other STIs. Chi-Square tests were used to compare the prevalence of (1) HIV and current STIs between MSM and MSW and (2) current STIs between people living with HIV and people not living with HIV. Logistic regression models were constructed to assess whether or not history of STIs were associated with current HIV infection.

**Results:**

The prevalence of HIV (9.2%), syphilis (7.2%), hepatitis B (2.7%), and high-risk anal HPV (8.4%) and penile HPV (21.3%) infections were high in Black men (*N* = 487) and were significantly increased in Black MSM compared with MSW; the prevalence of syphilis and high-risk HPV were also increased in men living with HIV. Men with a history of syphilis (OR = 6.48, 95% CI: 2.68,15.71), genital warts (OR = 4.32, 95% CI: 1.79,10.43) or genital ulcers (OR = 21.3, 95% CI: 1.89,239.51) had an increased odds of HIV infection.

**Conclusions:**

The HIV/STI prevalence was high among this sample of Black men, although the study design may have led to oversampling of men living with HIV. The associations between STIs and current HIV infection highlight the need for integrated of HIV/STI screening and treatment programs for Black men. Public health strategies are also needed to reduce disproportionate HIV/STI burden among Black MSM—including improving HPV vaccine coverage.

## Background

Over the past several decades, effective ways to prevent human immunodeficiency virus (HIV) infection and other sexually transmitted infections (STIs), as well as improved medical management for people living with HIV (PLHIV), have become widely available in high-income countries. In Canada, disproportionately high HIV and STI burdens exist among several subpopulations, including African, Caribbean, and Black (Black) communities, men who have sex with men (MSM), injection drug users (IDUs), and indigenous peoples [1]. In 2011, 208 people per 100,000 (0.2%) were infected with HIV in Canada [1]. However, Ontario’s Black population (3.9% of the total population) comprises approximately 22.5% of people living with HIV in the province. Furthermore, the HIV prevalence among Black men living in Ontario is six-fold higher than non-IDU White men [2, 3].

Although estimates of the prevalence of STIs exist for the general Canadian and Ontario populations, as well as some subpopulations (e.g. MSM), there are limited data available regarding the prevalence of STIs in Black communities. One recent clinic-based study assessed the prevalence of multiple STIs in a cross-sectional sample of Black women from the Greater Toronto Area, finding that viral STIs (e.g., herpes, cytomegalovirus [CMV], and human papillomavirus [HPV]) were far more common than in the general population [4–8]. Additionally, while not an STI, bacterial vaginosis is more prevalent among African-American women and is itself a potential risk factor for HIV acquisition and transmission [9–11].

There are several biological factors, including STI co-infections and altered levels of immune activation, which predispose individuals to HIV infection [2, 12]. These biological factors are believed to impact the two key determinants of HIV transmission risk: the HIV viral load in the PLHIV’s blood and genital secretions, and the number and density of HIV-susceptible cells in the sex partner (generally within their penile, vaginal, or anal mucosa) who is a person not living with HIV (non-PLHIV) [13, 14]. Both gonorrhea and the local reactivation of CMV increase HIV levels in the semen of a male PLHIV by approximately 0.7 log_10_ copies/mL and 1.0 log_10_ copies/mL respectively, [15, 16] where an increase of 1.0 log_10_ copies/mL is expected to double the risk of HIV transmission [17]. Co-infections in the HIV-negative partner also increased HIV susceptibility, including concurrent herpes simplex virus (HSV)-2 infection (a three-fold increase) [18] and bacterial vaginosis (a 60% increase in affected females) [11]. Despite evidence indicating increased susceptibility to HIV infection with concurrent STIs, these underlying mechanisms are unrelated to ethnicity and do not account for the discrepancies seen between Black and other communities.

Social factors also play a central role in HIV disease transmission and STI co-infection. There is extensive research indicating that social network mixing patterns are major contributors to racial/ethnic disparities in HIV infection [19–22]. Most of the available data are based on samples from populations in the United States. Several key studies indicate that the closed characteristics of social/sexual networks increase the odds that Black/African-Americans will be exposed to HIV or another STI [20–23]. Other studies indicate that disparities in the distribution of social determinants (e.g., income, incarceration, homophobia) influence between-group and within racial-group disparities in HIV incidence [19, 24–26]. A recently published study also found that partner-mixing patterns and recreational drug use were association with STI co-infection among MSM living with HIV in Canada [27].

To date, there is a very limited body of information regarding the prevalence of STIs and HIV co-infections in Black communities in Canada, particularly among Black men [28–34]. Black men, specifically MSM are disproportionately burdened with a high prevalence of HIV [1, 34, 35] compared to the general population. Despite these alarming rates, data regarding the prevalence of other co-infections in Black men, and the impact of these infections in Black MSM compared to men who only have sex with women (MSW), are limited. The main purpose of the Kali (Swahili for “fierce” or “hot”) study was to investigate the prevalence of HIV, STIs, and HIV/STI co-infections in a community-recruited, non-probability sample of Black men from Toronto, Ontario, Canada. A secondary purpose of the study was to examine whether past histories of STIs were associated with current HIV infection. The study also assessed whether HIV and STI prevalence, as well as associations between STI histories and current HIV infection, differed between Black MSM and MSW.

## Methods

### Design and setting

A cross-sectional descriptive epidemiological study was conducted with Black men from across the City of Toronto between 2011 and 2013. Toronto is the fourth largest city in North America and the largest city in Canada by population size. Toronto is also home to the largest Black community in Canada [3]. The Kali study was conducted at community health centre (CHC) sites in the downtown (Women’s Health in Women’s Hands CHC), eastern (Taibu CHC) and western (Rexdale CHC) corridors of Toronto. These three CHC sites were selected based on their geographic locations, to offer participants multiple options to access the study. Additionally, these specific clinic sites all had strong reputations for providing multi-culturally responsive health services to individuals from Black communities. The study was approved by the University of Toronto HIV Research Ethics Board.

### Recruitment and informed consent

Black men, selected from the greater Toronto community, were hired as peer recruiters and trained to engage in community-based outreach to identify potential study participants. Peers were selected based on their reputation within their communities, and to reflect ethnic and geographic (e.g., Toronto East, Toronto downtown, York, Scarborough) diversity of the greater Toronto area. The peer-based recruitment strategy used multiple peer-led approaches to identify Black men for potential enrollment in the study. To identify men who were interested to enroll in the study, peer-recruiters used their social network contacts to spread the word about the study and engaged in direct street-outreach within their neighborhoods (*n* = 140). After first obtaining permission from potential recruits, the peer recruiters documented the recruits’ name and the contact information on a spreadsheet that was submitted weekly to the research office. A trained research assistant-initiated follow-up recruitment calls to the names on the contact spreadsheets. The study also relied on word-of-mouth referrals of people interested to participate. Word-of mouth referrals are those that were not the result of active recruitment by peer-recruiters, but that resulted from the men hearing of the study from previous study participants (*n* = 182), or who heard about the study from family/friends who were aware of the study but were not previously enrolled (*n* = 114). A few men enrolled in the study after contacting the office in response to seeing posters (*n* = 8) or flyers/promotional cards (*n* = 15) advertising the study. A small number of men (*n* = 28) who enrolled in the study did not report the method by which they learned about the study.

Informed consent was obtained from each participant by the research coordinator. The research coordinator was present at each study site for the participants’ scheduled enrollment visits. Prior to enrollment, all potential participants were provided a copy of an information and consent form that described the specific activities in which they were being asked to participate. The research coordinator reviewed the form and invited the potential participants to read along, if desired, to ensure that each person clearly understood the purpose of the study and the process. The research coordinator also described potential risks of study participation and the strategies that were taken (e.g. secure online data entry, confidentiality training for RAs) to ensure privacy and confidentiality. The coordinator also emphasized that the study was voluntary and they were free to decline participation in any or all components of the study. Throughout the informed consent process, participants were encouraged to ask questions. After the coordinator answered questions to the participants’ satisfaction, they were asked to sign the form indicating their informed consent to participate in the study.

### Data collection

Data collection took approximately 60–90 min to complete for each participant. All data collection took place within one of the three designated CHC study sites. Participants had the option to choose which of the three CHC sites they visited for study enrollment and data collection. Survey data were collected using an audio computer-assisted self-interview (ACASI). The RA then provided the participants with step-by-step instructions on how to complete the questionnaire on the computer using ACASI. Care was taken during the interview design to ensure that the reading language of the survey was accessible to those with a Grade 5 reading level. The study RA was available to assist the participant with any questions or technical difficulties that he may have encountered during the self-interview. The RA was also available to administer the survey to a participant if, for whatever reason, he decided that he did not want to self-administer.

Study participants also provided biological specimens for clinical diagnostics. Two 10 ml vacutainer tubes of blood were collected by venipuncture, and a 50 ml first void urine sample was self-collected by the participant. Men were also provided with instructions for the self-collection of a saline-moistened penile swab (penile shaft and prepuce) for molecular HPV diagnostics, as well as an optional anal swab for HPV. All specimens were stored on location at CHCs for up to 16 h before being transferred by a laboratory assistant to the University of Toronto where the specimens were delivered to designated laboratories for processing.

### Measures

The ACASI survey was used to capture social and behavioral variables to more fully characterize the sample. These variables included age, education, income, marital status, sexual orientation, languages spoken, region of birth and immigration status. Participants self-reported their STI histories by responding “yes” or “no” to indicate whether a healthcare provider had ever told them that they had a chlamydia, gonorrhea, syphilis, genital herpes, HPV infection or if they have ever had genital ulcers. Participants also had the option to indicate if they “forgot the name of the STI.” They could also indicate if they were infected with an STI other than the ones presented to them on the survey list, but they were not otherwise asked to specify the name of the STI. The MSM category was created by coding any case where a man reported ever having sex with another man, inclusive of men who also had a history of sex with women. All but two of these coded cases also reported having sex with men in the previous 6 months. Men who reported a history of sex with women but never reported a history of sex with men were coded as men who have sex only with women (MSW). Biological specimens were collected for laboratory testing of HIV, chlamydia, gonorrhea, hepatitis B (HBV) and C (HCV) viruses, human papillomavirus (HPV), cytomegalovirus (CMV), herpes simplex virus (HSV) 1 and 2.

### Laboratory methods

Serum enzyme immuno-assays (EIA) were performed for HIV, CMV, HSV-1, HSV-2 and syphilis. Informed consent was obtained from each participant to screen their serum specimen for HIV. Participants were provided with pre- and post-test counseling for HIV. All reactive HIV results were confirmed by Western blot. Serologic testing was also performed to detect HBV surface and core antigens and HBV and HCV antibodies. Reactive HCV tests were confirmed using Bio-Rad Monolisa anti-Hep C Plus Version 2. A nucleic acid amplification test (NAAT) was used to detect *Neisseria gonorrhoeae* and *Chlamydia trachomatis* infection (Becton Dickinson ProbeTec nucleic acid amplification and detection system). HPV was diagnosed through self-administered anal and penile swabs that were analyzed using polymerase chain reaction (PCR; Roche linear array). The HPV assay used detected the primary high-risk oncological subtypes, HPV 16 and 18, which have been shown to be present in up to 87% of HPV-positive invasive anal cancers [36]. Participants were informed of their laboratory results and referred to treat of any newly diagnosed infection.

### Statistical analysis

Prevalence was calculated separately for MSM and MSW. Chi-square tests were used to compare the prevalence of current laboratory confirmed HIV and STIs in MSM with that of MSW. Correlations between all study laboratory confirmed variables were assessed using the phi coefficient for binary outcomes. Comparisons on the prevalence of current laboratory confirmed STIs between PLHIV and non-PLHIV men were also made using Chi-square tests. Due to large number of HPV subtypes, the anal and penile HPV variables were recoded as “any high-risk HPV-anal HPV” or “any high-risk HPV-penile” if any high-risk oncological subtype (HPV 16 or 18) was detected. Logistic regression models were used to examine whether histories of STIs were associated with HIV infection and whether the effect was consistent for MSM and MSW. Nine separate models were fitted for history of 9 different STIs, group (MSM vs. MSW), and STI x group interaction. The dependent variable was the HIV infection status. For those that did not have a significant interaction, the interaction term was removed and the main effect of each STI variable on HIV infection was tested, adjusting for MSM. No other sociodemographic variables were controlled in the logistic regression models. All analysis was conducted using SAS 9.3 (SAS, Cary, NC).

## Results

### Study population

Demographic data are presented in Table 1. The mean age of the men in the study was 36 years (*SD* = 13.75). The average age of those men born outside of Canada (*n* = 315; 64.68%) at arrival in Canada was 19.58 years (*SD* = 12.48 years) and they subsequently lived in Canada for an average of 23.4 years (*SD* = 13.66 years). Most men in the sample (*n* = 395; 82%) have only ever had sex with women with a small proportion of the sample (*n* = 86; 18%) reporting that they ever had sex with another man. Most MSM in the sample self-identified as homosexual (*n* = 82; 95%). A significantly higher proportion of MSM (37% v. 11%) were refugee claimants on first arrival to Canada, while more MSW (41% v. 21%) were landed/permanent residents on first arrival in Canada.

**Table 1:** Demographic characteristics among Black men who have sex with men (MSM) and Black men who only have sex with women (MSW)

Characteristic	Total	MSM	MSW
Total participants	487	86	395
Age (years)*			
Mean	36	37	36
Median (IQR)	34 (24–46)	35 (27–46)	34 (22–47)
16–24	29	17	32
25–34	21	29	19
35–44	20	21	20
45 or older	30	33	29
Education (%)**			
Less than secondary school	6	6	6
Some/completed secondary school	50	35	53
Some/completed college/university	41	52	38
Some/completed graduate education	4	7	3
Marital status (%)			
Married/Common-law	14	15	13
Separated/divorced/widower	14	12	14
Single	73	72	71
Annual Household Income (%)			
Less $10,000	42	37	44
$10,000 - $19,999	21	26	20
$20,000- 49,999	18	22	17
$50,000 or more	2	5	2
Don’t know	7	6	7
Declined not answer	10	5	11
Language spoken (%)			
N	16	15	15
English	79	79	76
French	1	0	1
Spanish	0	0	1
Other	4	3	4
Region of Birth (%)***			
Africa	26	16	28
Caribbean	35	58	30
Canada	35	21	39
Other	4	5	3
Status at first arrival in Canada (%)***			
Landed/permanent resident	58	21	41
Refugee claimant	25	37	11
Temporary worker	1	2	0
Visitor	8	12	3
Student	5	5	3
Non-status	4	2	2

Note: *p < .05, **p < .01, ***p < .00. Note: Total sample size was 487, and six subjects refused to report whether they ever had sex with men

### Prevalence of HIV and STIs: comparisons by sexual behavior category

The prevalence of HIV infection was 9.2% (Table 2). The HIV prevalence among MSM (38%) was significantly higher than among MSW (3%). MSM and MSW did not have any difference in the prevalence of bacterial STIs with the prevalence of chlamydia and gonorrhea being less than 5% in both groups. There were no cases of gonorrhea detected in MSM. Over 10% of the men in the sample (7.2%) were diagnosed with syphilis. A significantly higher proportion of MSM were diagnosed with syphilis than were MSW. There were no differences in proportions of HCV, HBV and HSV-2 between MSW and MSM, although more MSM were diagnosed with other viral infections. There were no differences in proportion of chlamydia diagnoses between PLHIV and non-PLHIV men (Table 3). The prevalence of syphilis was significantly higher in PLHIV men compared to non-PLHIV men. There was no group difference in the prevalence of HCV or HBV infection between PLHIV and non-PLHIV men. Significantly more PLHIV men were diagnosed with other viral infections compared to non-PLHIV men.

**Table 2:** Prevalence of Sexually Transmissible Infections, including HIV in non-probability sample of African, Caribbean and Black Men in Toronto, comparison between MSM and MSW (*N* = 481)

Current STI	Sexual Behavior Category		*χ*^2^ (1)
	MSM (*n* = 86)	MSW (*n* = 395)	
HIV	38.4	3.0	103.69***
Chlamydia	3.5	4.6	0.20
Gonorrhea	0	0.8	0.66
Syphilis	17.4	5.1	16.31***
Hepatitis B	5.8	2.0	3.75
Hepatitis C	5.8	3.5	1.00
Herpes Simplex Virus 1	82.6	71.4	5.02*
Herpes Simplex Virus 2	58.1	50.1	1.99
Cytomegalovirus	75.6	43.8	27.9***
Any high risk HPV- penile	32.6	19.2	6.56*
Any high risk HPV- anal	29.1	4.1	15.07***

Note: *STI* sexually transmitted infections, *HPV* Human papilloma virus

*p < .05, ***p < .001; Total sample size was 487, and six subjects refused to report whether they ever had sex with men

**Table 3:** Prevalence of Current Sexually Transmissible Infection among HIV Positive and HIV Non-Infected African, Caribbean and Black Men (*N* = 486)

Current STI	HIV Status		*χ*^2^ (1)
	HIV non-infected (*n* = 440)	HIV-positive (*n* = 46)	
Chlamydia	4.6	2.2	0.58
Gonorrhea	0.7	0	0.32
Syphilis	5.0	28.3	34.69***
Hepatitis B	2.3	6.5	2.71
Hepatitis C	3.64	6.5	0.99
Herpes Simplex Virus 1	70.9	93.5	10.42**
Herpes Simplex Virus 2	48.9	76.1	12.05**
Cytomegalovirus	45.2	91.3	34.87***
Any high risk HPV- penile	19.3	43.5	16.76***
Any high risk HPV- anal	4.8	43.5	29.31***

Note: *STI* sexually transmitted infections; **p < .01, ***p < .001; Total sample size was 487. HIV status not reported for one subject

### Self-reported STI history and the odds of current HIV infection

Syphilis was the only bacterial infection associated with increased odds of HIV infection. (Table 4). Genital warts (from HPV) and genital ulcers (from HSV-2) were associated with greatly increased odds of HIV infection. Self-reported history of having an “other STI” was associated with 20-fold increased odds of HIV infection. Similarly, having a history of STI with a forgotten name was associated with increased odds of HIV infection. Logistic regression models of current HIV infection, including interaction between sexual behavior (MSM vs. MSW) with self-reported STI history, did not reveal significant interactions with syphilis, genital warts, genital ulcers, or “STI, forgot name”. Self-reported history of “Other STI” and sexual behavior category (MSM v. MSW) did show significant interaction (*p* < .01) effects with “other STI” showing a stronger association with current HIV infection in MSW (OR: 79.93, 95% CI: 11.50–555.72) compared to MSM (OR = 2.93; 95%CI: 0.65, 13.32).

**Table 4:** Logistic Regression of STI History Associated with HIV Infection at Baseline (N = 486)

Past STI	*OR (95% CI)*	*p<*
Chlamydia	1.27 (0.51,3.18)	0.605
Gonorrhea	1.64 (0.77,3.47)	0.200
Syphilis	6.48 (2.68,15.71)	0.000
Genital warts	4.32 (1.79,10.43)	0.001
Genital herpes	3.49 (0.68,17.87)	0.133
Genital ulcers	21.25 (1.89,239.51)	0.013
STI, forgot name	5.3 (2.33,12.05)	0.000
Other STIs	20.41 (6.32,65.89)	0.000

Note: *STI* sexually transmitted infections. Total sample size was 487. HIV status not reported for one subject

## Discussion

The purposes of this study were to investigate the prevalence of HIV, STIs and HIV/STI co-infections and to investigate whether past or current STIs were associated with HIV infection in a community-recruited, non-probability sample of Black MSM and MSW in Toronto, Ontario, Canada. The prevalence of HIV in Black MSM was extremely high (38.4%). In fact, it is much higher than the HIV prevalence estimates for the general Black population in Ontario (18%) [37] and the general population of MSM in Ontario [35]. Black MSM also accounted for 93% of PLHIV men in the study sample. The prevalence estimate in this study was not generated from a probability-based sample; however, these data indicate a need for a larger population-based bio-behavioral survey that will allow for a proper estimation of HIV prevalence in Black men in Toronto and subsequent investigation of significant disparities between MSM and MSW. This is the first known report to provide evidence of sexual behavior group (MSW versus MSM) disparities in HIV prevalence between subpopulations of Black men in Toronto. Moreover, this data suggests greater attention must be given to allocating public health resources that support evidence-based strategies and clinical tools to reduce the prevalence of STIs among Black men, as one approach to reducing HIV disparities.

The prevalence of bacterial STIs such as chlamydia (4.1%) and gonorrhea (0.4%) in the sample was low; however, the prevalence of active syphilis infection (11.25%) was high. This may in part reflect an overall increase in syphilis incidence in Ontario, with significant increases in 2002 and 2009 [38]. Syphilis prevalence was significantly higher in MSM than in MSW (17.4% vs 5.1%). Syphilis prevalence among Black MSM in the sample was twice as high as the prevalence found in a recent study among a general sample (*N* = 442) of MSM in Toronto (17.4% vs. 7.2%) [39]. There was also a high prevalence of HBV, HCV, and HPV—with higher HPV prevalence in Black MSM than MSW. The high prevalence of vaccine preventable infections (i.e., HBV and HPV) in this sample of Black men signals a need to target earlier identification of male youth for vaccination in public health programs. Compared to a recently study of a clinic-based sample of MSM (Black and non-Black) in Toronto [39], the MSM in our study sample had a higher prevalence of chlamydia (3.5% vs. 0.6%), active syphilis (17.4% vs. 8.4%), HSV-1 (82.6% vs. 75.4%), HSV-2 (58.1% vs. 50.0%), active HBV infection (5.8% vs. 2.1%), and lower prevalence of gonorrhea (0.0% vs. 0.2%), HCV (5.8% vs. 8.0%), CMV (75.6% vs. 92.2%) and high risk anal HPV (29.1% vs. 62.2%) [39]. Although we found a higher prevalence of several STIs in our sample of Black MSM compared to the MSM sample in the study by Remis, et al. [39], neither study used a probability-based sample; therefore, we cannot eliminate the possibility that the findings do not reflect the STI epidemiology of the larger populations of MSM, including Black MSM. The lack of population-level data on viral and bacterial STI prevalence remains an important gap in understanding of the STI epidemiology among MSM—a high priority population for HIV prevention and treatment in Ontario.

The prevalence of syphilis and high-risk HPV were higher in PLHIV than in HIV non-PLHIV Black men. CHCs that target services to Black communities may consider integrating HIV/STI and viral hepatitis screening into health promotion services. It is important to recognize that targeting STI screening and treatment for PLHIV men poses challenges. Given the history of and ongoing stigma towards PLHIV within the healthcare system it may be difficult to convince people to regularly pursue STI screenings in environments where they feel unwelcomed. Furthermore, some PLHIV may perceive that targeted STI screening contributes to a narrative of promiscuity and insufficient prophylaxis amongst people with HIV. Therefore, while integrating STI screening and treatment for men living with HIV may play an essential role in reducing co-infection burden, particularly for syphilis, it should not be implemented in a punitive manner (e.g., verbal reprimands, choosing an intramuscular versus oral treatment delivery route). Laws that criminalize sexual behavior of men living with HIV are also punitive and may complicate screening efforts due to fear and stigma since Black men are disproportionally subjected to prosecution under these statutes [40, 41]. The high prevalence of HIV and STIs in the sample is an urgent finding that has immediate relevance for public health practice. Further interventions are essential but must abide by the bioethical tenet of “primum non nocere” or “first, do no harm”.

Self-reported histories of syphilis, genital warts and genital ulcers were associated with current seropositive HIV status in both MSM and MSW. Men with self-reported history of an STI with a name that could not be remembered were five times more likely to be a PLHIV than those who could name their past infection. Similarly, those with who reported that they had an “other STI” were 20 times more likely to be a PLHIV. A history of “other STIs” was positively associated with current HIV infection in MSM and MSW. Moreover, the association of self-reported history of “other STIs” with current HIV infection was stronger in MSW than for MSM. This result may reflect the intersection of clinical and social factors. It is not clear from the data whether the inability to recall the name of an STI demonstrates low health literacy, which may be a social factor that contributes to HIV vulnerability for Black men. There is a strong evidence indicating that, HIV/STI knowledge (e.g., prevention, transmission, treatment) is necessary, but insufficient on its own for enacting HIV risk reduction behaviors [42, 43]. Interventions that facilitate patients’ understanding of their diagnoses can promote their self-determination in healthcare encounters. This may be protective through increasing their understanding of their options and the logic underlying those options—improving their capacity to make informed decisions on developing a personalized HIV prevention plan [44–46].

Sexual health programs that screen for STIs may consider the potential benefits of incorporating syphilis screening for Black men. HIV pre-exposure prophylaxis (PrEP) is an evidence-based biomedical prevention approach [47–49] that could be targeted to non-PLHIV ACB men and to the non-PLHIV sexual partners of PLHIV Black men as a strategy to minimize their risk for HIV seroconversion. The Canadian Guidelines on HIV PrEP and Non-Occupational Post-Exposure Prophylaxis do not currently recommend PrEP for men who don’t have sex with men, even if they have a history of STI; nonetheless, the findings from this study suggests that Black men (including MSW) with history of syphilis may be a population to consider as possible candidates for HIV PrEP. Furthermore, Public Health Ontario reported in September 2015 that of all new cases of infectious syphilis reported between 2004 and 2015, 40% of individuals were co-infected with HIV (prior to or within one-year of syphilis diagnosis) [38]. Additionally, STI history—and particularly syphilis history—may be a consideration for high-frequency HIV testing as a strategy for early identification of HIV infection and rapid linkage to HIV care and treatment. While HIV prevention intervention strategies are traditionally based on immediate (or recent) behavioral risks, local and regional health units may also benefit from using local STI and HIV epidemiological data to target individuals who may be at high risk for HIV acquisition.

The results of the study should be considered in view of several important limitations. First, the study uses data from a non-probability of sample of Black men. The recruitment approach utilized multiple peer-based strategies and some participants contacted the study via passive-referral processes, the combination of which aimed to limit bias in the overall sample population. However, despite these efforts to maximize the diverse representation of the study sample, the findings here cannot be considered representative of the general population of Black men in the City of Toronto or the Province of Ontario. Nonetheless, the results provide preliminary evidence that can inform public health planning. For example, the epidemiological data on Black communities has historically been limited to people who immigrated to Canada from HIV-endemic counties in the sub-Saharan African and Caribbean regions, often excluding Black-identified men from non-HIV endemic regions such as Canada, United States, and United Kingdom. The data reported in this study include Black-identified residents of Toronto regardless of their country of origin. Future studies that include the broader range of Black-identified individuals in a probability-based sample will help to generate a more accurate estimate of HIV and STI prevalence among this population of men and thereby strengthen the evidence-base for policy and practice decisions. Future epi-surveillance studies can be enhanced with integrated components that focus on implementation of evidence-based HIV/STI prevention strategies as a tactic to maximize the use of scarce resources that will generate public health data while contributing to population impact. The results regarding the differential effect of self-reported history of an STI with a name that could not be remembered on the risk of current HIV infection, between MSW and MSM, should be viewed with caution because of small sample size which resulted in estimates with very wide confidence intervals.

## Conclusion

The HIV/STI prevalence was high among this community-based sample of Black men. Current and past STIs were associated with HIV infections, with disparities noted in MSM. STI screening and treatment among ACB men, including increased access to vaccines to prevent infections such as HBV and HPV, may help reduce their risk of HIV infection. The results have important implication for advancing approaches that integrate research and public health strategies to reduce disparities in HIV and STI infections in Black populations.

## Abbreviations

ACASI: Audio computer assisted self-interviews
ACB: African, Caribbean, Black
AIDS: Acquired immune deficiency syndrome
CHC: Community health centre
CMV: Cytomegalovirus
EIA: Enzyme immune-assays
HBV: Hepatitis B virus
HCV: Hepatitis C virus
HIV: Human immunodeficiency virus
HPV: Human papillomavirus
HSV: Herpes simplex virus
IDU: Injection drug users
IQR: Interquartile range
MSM: Men who have sex with men
MSW: Men who only have sex with women
NAAT: Nucleic acid amplification test
nPEP: Non-occupational post-exposure prophylaxis
PCR: Polymerase chain reaction
PrEP: Pre-exposure prophylaxis
RA: Research assistant
STIs: Sexually transmitted infections
